# Renin Trajectories and Outcome in Stable Heart Failure with Reduced Ejection Fraction (HFrEF) on Contemporary Therapy: A Monocentric Study from an Austrian Tertiary Hospital Outpatient Clinic

**DOI:** 10.1155/2023/8883145

**Published:** 2023-10-31

**Authors:** Emilie Han, Suriya Prausmüller, Annika Weidenhammer, Georg Spinka, Henrike Arfsten, Philipp E. Bartko, Georg Goliasch, Martin Hülsmann, Noemi Pavo

**Affiliations:** Department of Internal Medicine II, Clinical Division of Cardiology, Medical University of Vienna, 1090 Vienna, Austria

## Abstract

**Introduction:**

The renin-angiotensin system (RAS) is the main target of neurohumoral therapy in heart failure with reduced ejection fraction (HFrEF) effectively reducing mortality. Reasonably, renin might serve as a biomarker for risk prediction and therapy response. Renin indeed bears some additional value to clinical risk models, albeit the effect is not pronounced. Whether assessing renin trajectories can overcome the weaknesses of single renin measurements has not been reported.

**Methods:**

A total of 505 patients with stable HFrEF were enrolled prospectively and followed through routine clinical visits. Active plasma renin concentration was documented up to 5 years. Changes in renin were analyzed throughout the disease course, and survival was compared for different renin trajectories within the first year.

**Results:**

Baseline renin levels were not related to all-cause mortality (crude HR for an increase of 100 *μ*iE/ml: 1.01 (95% CI: 0.99–1.02), *p* = 0.414) but associated with unplanned HF hospitalizations (crude HR: 1.01 (95% CI: 1.00-1.02), *p* = 0.015). Renin increased during the disease course from baseline to 1-year and 2-year FUP (122.7 vs. 185.6 *μ*IU/ml, *p* = 0.039, and 122.7 vs. 258.5 *μ*IU/ml, *p* = 0.001). Both survival and unplanned HF hospitalization rates were comparable for different renin trajectories at 1-year FUP (*p* = 0.546, *p* = 0.357).

**Conclusions:**

Intriguingly, renin is not a good biomarker to indicate prognosis in HF, while renin trajectories over a 1-year period do not have an additional value. Rapid physiologic plasma renin variations, but also opposing effects of angiotensinogen-derived metabolites under presence of RAS blockade, might obscure the predictive ability of renin.

## 1. Introduction

The neurohumoral concept of heart failure (HF) was introduced in the early 1990s after the SOLVD and CONSENSUS trials have been published [1]. Since then, neurohumoral activation is acknowledged as a hallmark of HF driving disease progression. The renin-angiotensin system (RAS) is the most known neurohumoral system affected in HF and the most current effective pharmacological interventions recommended by the guidelines for HFrEF target or at least impact the RAS [2].

Renin catalyzes the formation of angiotensin I (AngI) from angiotensinogen, which is the rate-limiting step of the angiotensin cascade. Angiotensin II (AngII) is then generated from AngI through angiotensin-converting enzyme (ACE) and is thought to be the main effector peptide responsible for deleterious cardiovascular effects as vasoconstriction, inflammation, fibrosis, and hypertrophy mediated through the AngII type 1 receptor (AT1R) [3]. AngII is also one of the main regulators of aldosterone release from the adrenal cortex. Aldosterone mediates its effects by binding to the mineralocorticoid receptor (MR) on the epithelial cells of the renal cortical collecting duct. It increases the amount of apical epithelial sodium channels resulting in increased sodium reabsorption and potassium excretion and influencing body fluid balance [4]. Aldosterone promotes myocardial fibrosis probably facilitating adverse remodeling. RAS inhibitors are the most successful agents blocking the actions of AngII either via ACE inhibition or by inhibition of AT1R and represent the main pillar of current treatment of HFrEF together with MR antagonists (MRA) and sodium-glucose cotransporter-2 inhibitors (SGLT2i) and beta blockers. Nevertheless, disease progression in HFrEF cannot be halted, and ongoing RAS actions have been suggested as a trigger. Several mechanisms have been proposed for continuing AngII actions despite assumed optimal RAS blockade, such as increased renin activity based on AngII negative feedback loop or escape mechanisms through alternative molecular regulation [5]. Disappointingly, direct renin inhibition by aliskiren, although effectively lowering plasma renin activity [6], did not modify outcome in a large randomized trial [7].

The assessment of RAS activity, especially under treatment, may offer the possibility to identify incomplete RAS inhibition or activated escape mechanisms and thereby fine-tune pharmacological RAS blockade as a personalized approach. Although this strategy seems temptingly logic, simple, and straightforward at first, the investigation of RAS activation in HFrEF under treatment has been addressed by relatively few studies. Consequently, renin levels are currently neither used for risk stratification nor for an individualized approach for optimizing RAS blockade. In CONSENSUS, higher AngII was observed in patients with unfavorable outcome and the reduction of 6-month mortality by enalapril was more pronounced in patients with AngII above the median [8]. A substudy of the Val-HeFT trial investigated the effect of renin and other neurohumoral markers on HF progression [9]. Renin above the median was associated with higher mortality rates compared to lower renin levels, yet the additional prognostic value of renin when combined upon a clinical model with BNP was nonsignificant for mortality and morbidity [10]. Another study showed that renin activity was an independent predictor of death, yet if N-terminal pro-B-type natriuretic peptide (NT-proBNP) levels were low, patients had a comparably good prognosis independent from renin levels [11].

In contrast to single renin measurements, the evolution of renin levels throughout the disease course has not been investigated yet. Serial renin measurements may overcome the prognostic weaknesses of single renin values by addressing the disease progression individually. The aim of this study was to investigate the changes of renin levels during the course of HFrEF and the impact of different renin trajectories on patient outcomes.

## 2. Methods

### 2.1. Study Population and Study Endpoint

Consecutive adult patients with chronic stable HFrEF on guideline-directed medical therapy (GDMT) were enrolled from the prospective registry of the HF outpatient unit at the Vienna General Hospital, a university-affiliated tertiary care center, between June 2013 and August 2021. Inclusion criteria were as follows: an age ≥ 18 years, the diagnosis of HFrEF, defined as a history of signs and symptoms of HF and formerly documented left ventricular ejection fraction (LVEF) below 40%, medical therapy according to current HF guidelines [12], clinically stable HF at presentation in our outpatient unit, and available active plasma renin concentration (ARC) measured while sitting upright. Exclusion criteria comprised of patients with solid or hematological cancers undergoing active antineoplastic therapy up to three months before study inclusion, symptomatic infectious disease, phaeochromocytoma, use of oral contraceptives, and patients with decompensated HF at the time of inclusion. Clinical, imaging, and laboratory parameters were documented at baseline and consecutive routine visits. Change of HF (beta blockers, RAS inhibitors (angiotensin-converting enzyme inhibitors (ACEi), angiotensin receptor blockers (ARBs), and angiotensin receptor-neprilysin inhibitors (ARNI)), MRA, and SGLT2i) was assessed for all patients between baseline and 1-year FUP, which was defined as an addition or withdrawal of a drug [12]. Data on all-cause mortality and unplanned HF hospitalizations served as outcome parameters. All-cause mortality data were provided by the Austrian Death Registry.

### 2.2. Laboratory Measurements

Routine laboratory parameters including NT-proBNP and plasma aldosterone were measured by specific immunoassays according to standard laboratory procedure at first presentation. Active plasma renin concentration (ARC) was similarly measured by the standard laboratory procedure using a chemiluminescence assay (DiaSorin Liason XL). The analytical limit of detection was 0.5 *μ*IU/ml, while functional was 1.6 *μ*IU/ml and coefficient of variation was reported as 6.6% (27.5 *μ*IU/ml) and 9.8% (97.2 *μ*IU/ml).

To assess the course of renin levels over time, renin levels were documented for all patients at baseline and at available follow-up (FUP) visits 12 ± 6 months, 24 ± 6 months, 36 ± 6 months, 48 ± 6 months, and 60 ± 6 months, whenever available. In case of multiple measurements, the closest values to index date were entered. Renin levels were regarded as normal if ≤46 *μ*IU/ml (measured while sitting upright) and termed elevated otherwise.

### 2.3. Statistical Analysis

Categorical variables were compared using chi-square test, while continuous variables were compared using the Kruskal-Wallis or Mann–Whitney *U* test. Testing for normality was done using the Shapiro-Wilk test. Baseline renin levels were correlated with NT-proBNP by calculating Spearman's rho correlation coefficient (*ρ*). The Cox proportional hazard regression analysis was used to evaluate the association between renin and outcome measures and given as hazard ratios (HR), 95% CI. The Kaplan-Meier plots were generated, and groups were compared by log-rank test. Renin levels between baseline and FUP timepoints were compared by nonparametrical unpaired tests. To assess the effect of changes in renin levels, patients were categorized into four groups based on renin trajectories between baseline values and first year of observation ((1) normal-normal = normal renin at BL and 1-year FUP; (2) elevated-normal = elevated renin at BL, normal renin at 1-year FUP; (3) normal-elevated = normal renin at BL, elevated renin at 1-year FUP; and (4) elevated-elevated = elevated renin at BL and 1-year FUP). Furthermore, renin levels were compared for all patients who had three consecutive yearly measurements available leading up to the timepoint of last seen or death using the Kruskal-Wallis test. All statistical analyses were performed using R, version 4.0.5 (R Foundation for Statistical Computing, Vienna, Austria). A two-tailed *p* value < 0.05 was considered significant.

## 3. Results

### 3.1. Baseline Characteristics of the Study Population

A total of 505 HFrEF patients were included in the study. Baseline demographic, clinical, and laboratory characteristics are presented in Table 1. Median age was 62 years (Q1–Q3: 52–72), and 390 patients (77%) were male. Median NT-proBNP was 1783 pg/ml (Q1–Q3: 802–3632). A total of 64 patients (13%) were in New York Heart Association (NYHA) I, 246 patients (50%) in NYHA II, and 185 patients (37%) in NYHA III/III+. Heart failure medication was well established at study initiation with 457 (90%), 467 (94%), 375 (76%), and 31 (20%) patients receiving RASi, beta blockers, MRA, and SGLT2i, respectively. Of those patients with RASi, 238 patients (52%) received ACEi, 97 (21%) received ARB, 110 (24%) received ARNI, and 12 patients (3%) received ACEi and ARB in combination based on MRA intolerance. Additionally, around half (49%) of the study population received loop diuretics. Furthermore, 73%, 80%, and 97% of patients received a minimum of 50% of the recommended target dose for RASi, beta blockers, and MRA, respectively.

Renin values were 123 *μ*IU/ml (Q1–Q3: 27–621) and ranged from 0.8 to 42.9 *μ*IU/ml for the low, 43 to 339 *μ*IU/ml for the medium, and 340 to 9564*μ*IU/ml for the high tertile. Higher renin values were observed in patients with proportionally decreasing systolic blood pressure (*p* < 0.001), decreasing sodium concentrations (*p* < 0.001), more frequent MRA (*p* = 0.002) and loop diuretic (*p* = 0.009) use, and increasing aldosterone levels (*p* < 0.001). The majority of baseline parameters were similar between tertiles of renin tertiles.

### 3.2. Renin Distribution and Association of Renin with HF Severity and HF Medication

**Figure 1:**
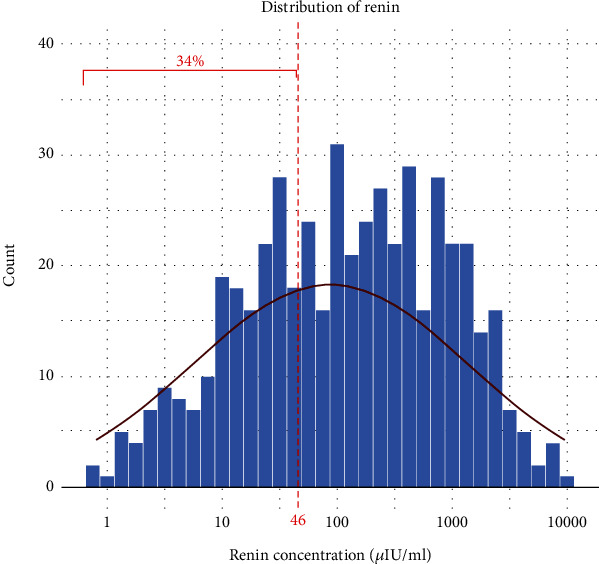
Baseline renin distribution. The distribution of renin is visualized as a histogram, 34% of patients had normal renin values as defined by laboratory cut-off, and normal distribution is additionally displayed.

Baseline renin levels showed a nonnormal distribution (*p* < 0.001) (Figure 1). Overall, 34% of patients showed normal renin levels despite HF and established HF therapy.

**Figure 2:**
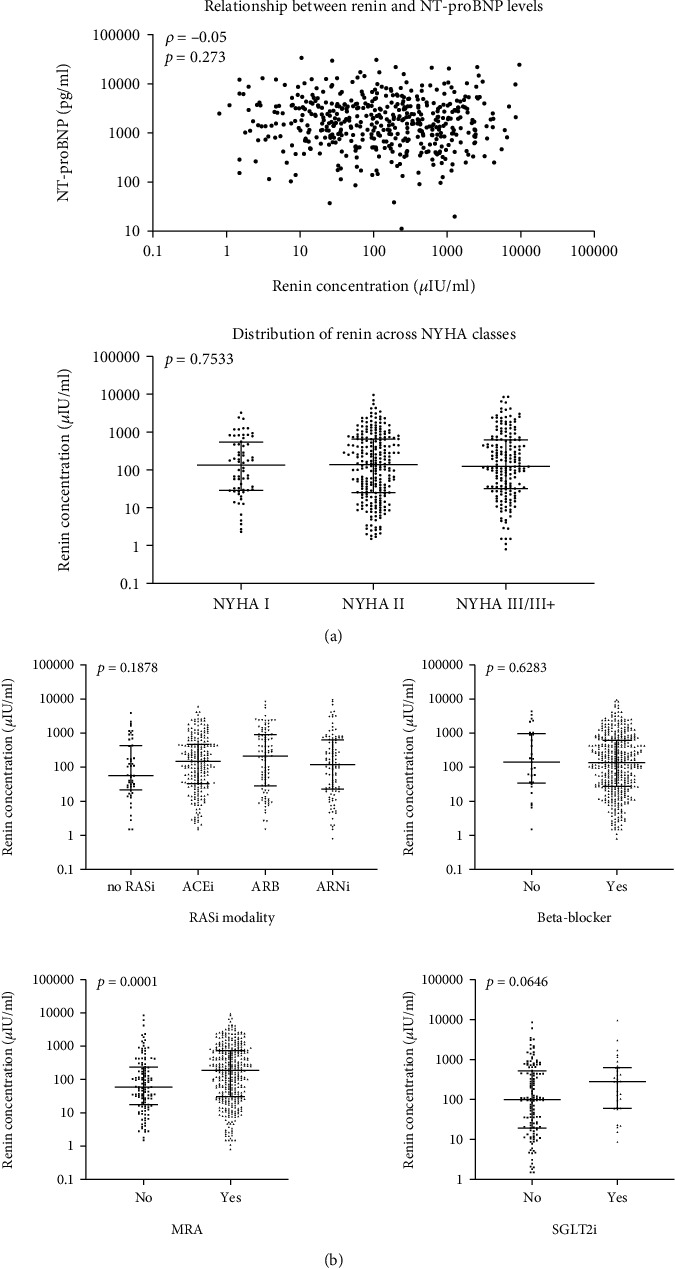
Baseline renin distribution and association of renin with HF severity and HF medication. (a) The association of renin with HF severity reflected by NT-proBNP levels and NYHA class is visualized for individual values as a scatter plot and Tukey's boxplots. The Spearman correlation coefficient for the former and level of significance are indicated. (b) Comparison of renin levels between different patients with any, with different, or without HF-specific medications. Individual values are shown alongside Tukey's boxplots. For all comparisons, nonparametrical tests (Mann–Whitney *U* and Kruskal Wallis) were used. *p* < 0.05 were regarded as significant.

Renin levels showed no relationship with HF severity reflected by NT-proBNP (*ρ* = −0.05, *p* = 0.273) and NYHA groups (NYHA I: 133.7 *μ*IU/ml (Q1–Q3: 29.6–498.3) vs. NYHA II: 127.9 *μ*IU/ml (Q1–Q3: 23.9–644.9) vs. NYHA III/III+: 122.7 *μ*IU/ml (Q1–Q3: 31.9–618.8), *p* = 0.753) (Figure 2(a)).

Renin levels were comparable for patients on different RAS inhibitors (no RASi: 57.0 *μ*IU/ml (Q1–Q3: 24.5–426.6) vs. ACEi: 133.1 *μ*IU/ml (Q1–Q3: 30.9–465.9) vs. ARB: 212.5 *μ*IU/ml (Q1–Q3: 28.9–916.1) vs. ARNI: 120.4 *μ*IU/ml (Q1–Q3: 23.2–627.0), *p* = 0.188) and patients with and without beta blockers (141.4 *μ*IU/ml (Q1–Q3: 35.9–916.1) vs. 125.9 *μ*IU/ml (Q1–Q3: 27.35–603.5), *p* = 0.6283) (Figure 2(b)). Higher renin levels were observed in patients with MRA (183.9 *μ*IU/ml (Q1–Q3: 28.8–714.8) vs. 59.3 *μ*IU/ml (Q1–Q3: 17.9–229.0), *p* = 0.001) (Figure 2(b)). Patients who were on therapy with SGLT2i at baseline had numerically higher renin levels, though the difference was statistically not significant (280.1 *μ*IU/ml (Q1–Q3: 62.4–624.5) vs. 99.9 *μ*IU/ml (Q1–Q3: 19.8–471.3), *p* = 0.065) (Figure 2(b)). Furthermore, the use of loop diuretics was associated with higher renin concentrations (94.0 *μ*IU/ml (Q1–Q3: 23.6–408.0) vs. 199.0 *μ*IU/ml (Q1–Q3: 34.8–702.0), *p* = 0.0030) (Figure 2(b)).

### 3.3. Baseline Renin Levels and Outcome

**Figure 3:**
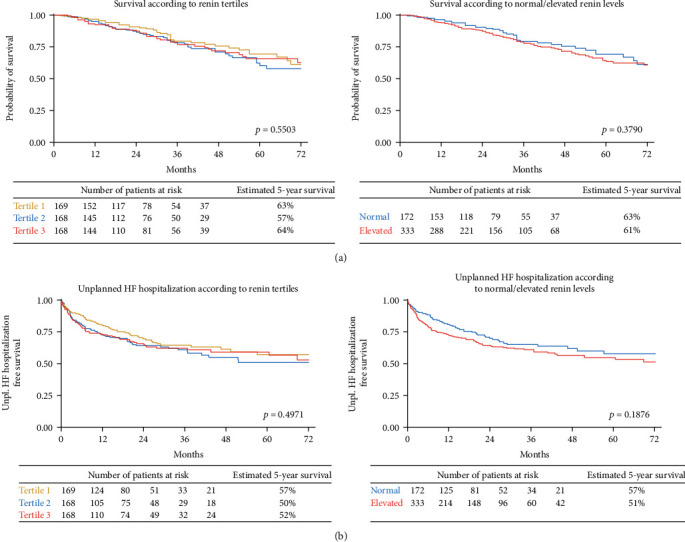
Survival according to baseline renin concentration. The Kaplan-Meier plot displays overall survival for patients with (a) normal vs. elevated renin and (b) according to renin tertiles. The difference between groups was assessed by the log-rank test.

In total, 172 (34%) patients had normal renin at baseline. Median FUP time regarding all-cause mortality was 34 months (Q1–Q3: 19–55), and from the total cohort, 123 (24%) patients died. The median time to unplanned HF hospitalization was 7 months (Q1-Q3: 2-17), and 181 (35.8%) patients experienced an event during follow-up.  Table 2 shows the crude and multivariable Cox regression analyses for baseline renin levels for all-cause mortality and unplanned HF hospitalization. Subgroup analysis is provided in Supplementary Figure 1. Baseline renin levels were not associated with all-cause mortality (crude HR for an increase of 100 *μ*iE/ml 1.01 (95% CI: 0.99–1.02), *p* = 0.414). This was congruent with comparable survival curves for different renin tertiles (*p* = 0.5503, log-rank test) and for normal vs. elevated renin levels (*p* = 0.3790, log-rank test) in the Kaplan-Meier analysis (Figure 3(a)).

Baseline renin levels were a risk factor for unplanned HF hospitalizations (crude HR for an increase of 100 *μ*iE/ml 1.01 (95% CI: 1.00–1.02), *p* = 0.015), which remained significant for adjusted models I (age, sex, and BMI) and II (model I + additional adjustment for comorbidity type 2 diabetes, coronary artery disease, and hypertension), but not model III (NT-proBNP, NYHA class, and aldosterone). No differences were seen in the Kaplan-Meier analysis across renin tertiles (*p* = 0.4971, log-rank test) or normal vs. elevated renin levels (*p* = 0.1876, log-rank test) (Figure 3(b)).

### 3.4. Evolution of Renin with HFrEF Disease Progression and the Impact of Renin Trajectories

**Figure 4:**
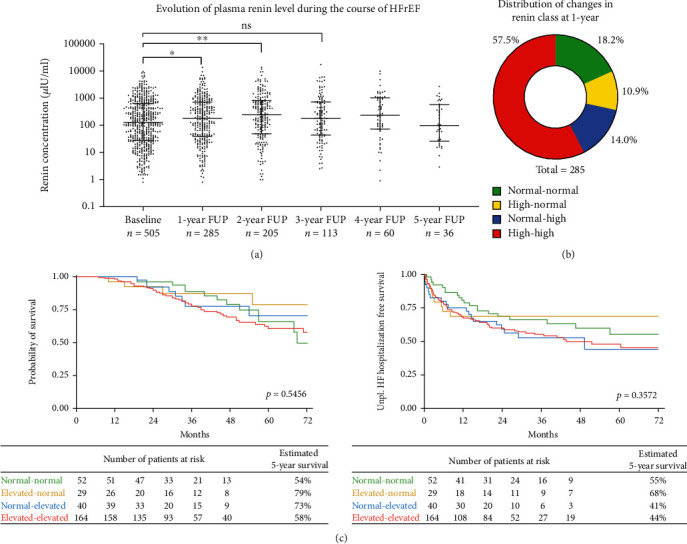
Evolution of renin levels and survival according to delta renin. (a) Evolution of plasma renin levels, baseline to 5-year follow-up. Individual values are shown alongside Tukey's boxplots. For comparisons between groups, the Kruskal-Wallis test was used. *p* < 0.05 was regarded as significant. (b) Distribution of changes in renin class at 1 year is visualized by donut chart. (c) The Kaplan-Meier plot displayed overall survival for the different renin trajectory groups according to changes of renin class at 1 year. The difference between the groups was assessed with log-rank test.

An increase in renin was apparent between baseline and 1-year FUP (122.7 *μ*IU/ml (Q1–Q3: 27.1–620.7) vs. 185.6 *μ*IU/ml (Q1–Q3: 41.0–723.3), *p* = 0.039) and baseline and 2-year FUP (122.7 *μ*IU/ml (Q1–Q3: 27.1–620.7) vs. 258.5 *μ*IU/ml (Q1–Q3: 49.0–808.1), *p* = 0.001), and a trend for higher renin levels was observed between baseline and 3-year FUP (122.7 *μ*IU/ml (Q1–Q3: 27.1–620.7) vs. 180.1 *μ*IU/ml (Q1–Q3: 44.5–713.4), *p* = 0.095) (Figure 4(a)).

Baseline and 1-year renin level pairs were available for a total of 285 patients. At 1-year FUP, 272 (95%), 281 (99%), 259 (90%), and 36 (34%) patients received medical therapy with RASi, BB, MRA, and SGLT2i, respectively. A minimum of 50% of the recommended target dose was achieved for 67%, 90%, and 76% of patients with RASi, beta blockers, and MRA, respectively. Changes in medication were performed in a total of 68 (24%) patients within 1 year. The change of renin within baseline and 1-year FUP between patients with change in HF therapy vs. patients without was similar (*p* = 0.497).

With 57.5%, the majority of patients showed elevated-elevated renin levels, while normal-normal, elevated-normal, and normal-elevated evolution were apparent for 18.2%, 10.9%, and 14.0% of patients (Figure 4(b)).  Table 3 shows HF-related baseline characteristics for these 4 groups. Patients in the elevated-elevated group had lower systolic blood pressure (*p* < 0.001) and lower sodium concentrations (*p* < 0.001) compared to other trajectories. This group was also characterized by more frequent ICD device therapy with 59% vs. 36%, 38%, and 42% for normal-normal, elevated-normal, and normal-elevated groups, respectively (*p* = 0.011). The majority of parameters, however, were similar between the four trajectories. Notably, renin trajectories were similar for patients with and without medication change within the first year of FUP (*p* = 0.257, chi-square test).

Table 2 shows the crude and multivariable Cox regression analyses for 1-year delta renin levels for all-cause mortality and unplanned HF hospitalization. 1-year delta renin was not associated with all-cause mortality or unplanned HF hospitalization.

Also, there was no difference regarding survival (*p* = 0.5456, log-rank test) or unplanned HF hospitalizations (*p* = 0.3572, log-rank test) according to different renin trajectories as shown by the Kaplan-Meier analysis (Figure 4(c)). The Kaplan-Meier analysis showed similarly comparable curves for both outcome measures when comparing 1-year delta renin values grouped by <50%, >50%, and ±50% or by renin elevation of more than 100 *μ*IU/ml vs. no increase at 1 year compared to baseline (Supplementary Figure 2).

**Figure 5:**
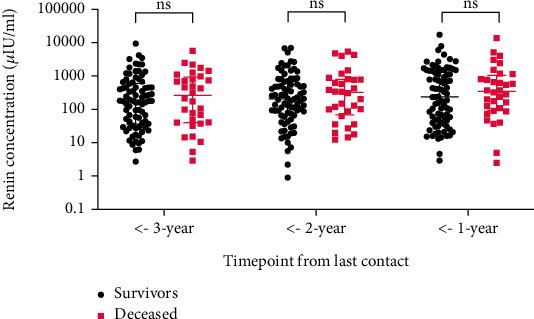
Comparison of renin levels of nonfatal and fatal cases of stable heart failure. Individual values are shown alongside Tukey's boxplots. For comparisons between groups, the Kruskal-Wallis test was used. *p* < 0.05 was regarded as significant.

Three consecutive yearly measurements up to the timepoint of last contact were available for 114 (22.6%) patients of the total cohort. Out of those patients, 32 (28%) were deceased, while 82 (72%) have survived until the censor date. Renin levels were not significantly different for patients with fatal outcome and survivors (Figure 5).

## 4. Discussion

This is the first study investigating the impact of renin trajectories in a relatively large cohort of HFrEF patients on timely GDMT. Despite advanced HFrEF with median NT-proBNP level of 1780 pg/ml and well-established GDMT, almost 34% of patients display renin levels within the normal range. Higher renin levels were associated with lower systolic blood pressure, lower serum sodium concentrations, higher aldosterone levels, and more frequent MRA use. Baseline renin was not associated with mortality in this cohort. Renin levels increased significantly during the course of HFrEF. However, different renin trajectories were not associated with mortality or unplanned HF hospitalizations. Similarly, there was no difference in renin levels within the last three years of contact between survivors and deceased patients.

According to the neurohumoral concept, HFrEF is associated with elevated neurohumoral markers including renin. Additionally, HFrEF therapy itself increases renin levels. ACEi and ARB have been reported to increase renin considerably, while diuretics, such as thiazides or potassium sparing diuretics, and calcium channel blockers also lead to an increase of renin, though at a lesser extent [13]. ARNI have been described to increase renin levels too [14]. Also, MRA were shown to cause dose-dependent renin increase compared with placebo [15]. Contrarily, the treatment with beta blockers in addition to RASi may lead to lower renin levels [16]. The population of this study was well treated with HFrEF medication, and in the dataset, renin was indeed higher for patients receiving MRA and diuretics. Data on the effects of SGLT2i on changes of renin are scarce. In a randomized controlled trial of hypertensive diabetic patients receiving a base therapy with RASi (ACEi or ARB), patients receiving dapagliflozin had significantly higher levels of plasma renin activity (PRA) than controls after 24 weeks of treatment [17]. Similarly, a strong trend could be observed for higher ARC in SGLT2i-treated patients in our cohort. Corresponding to renin's physiologic purpose, systolic blood pressure, plasma sodium concentration, and left ventricular function were found to be independent predictors of renin [18]. Also, in this dataset, patients with higher renin levels were more likely to have lower systolic BP and plasma sodium concentrations.

In patients who experience an acute HF hospitalization, HF severity was associated with more pronounced RAAS activation including higher renin and aldosterone levels [19]. Though aldosterone is influenced by current medical HF therapeutics, a subgroup of chronic HF patients still possesses aldosterone levels above the upper limits of normal even after complete blockage of RAS using ACEi [20].

Nevertheless, about a third (34%) of stable HFrEF patients still had normal renin levels. Earlier data investigating renin and the concentration of angiotensin metabolites in HFrEF showed that renin exerts an excellent direct correlation with AngII regardless of the mode of RAS blockade [18]. This suggests the presence of different renin phenotypes with on the one hand excessively activated RAS and ongoing deleterious AngII actions but on the other hand also patients with normal RAS activity despite HF and RAS blocker treatment. Whether this distinction is pertinent and could lead to a reasonable and effective therapeutic adaptation could be subject of future studies.

The prognostic ability of renin has been studied, albeit in few large-scale studies. The Val-HeFT trial with over 4200 patients found small additive prediction benefit of renin to clinical characteristics and NT-proBNP on mortality rates with limited clinical relevance [21]. A relatively small study with 293 included patients investigated chronic and acute decompensated HF patients on GDMT in 2017 showing that renin levels were higher in chronic HF compared to acute decompensated HF. Notably, here, prognosis was similar for different renin strata [22]. In another study [23], higher renin levels were associated with a higher rate of HF hospitalization or all-cause mortality, though without additive value in improving risk stratification of BIOSTAT-CHF prognostic models [24]. Other studies including up to 2913 patients have shown some prognostic ability of renin as it increases in cases of fatal compared to nonfatal acute decompensated HF [25, 26]. In this study, we could not observe a significant relationship between baseline renin levels and all-cause mortality. This could possibly be attributable to the size of our cohort, as most of the aforementioned studies demonstrating that an increase in renin is associated with increased risk have analyzed higher number of patients. It is therefore likely that very high renin concentrations are associated with augmented risk; however, this effect seems limited.

Contrasting with single renin measurements, natural evolution of renin and the impact of renin trajectories have not yet been investigated. Serial renin measurements in this dataset suggest that renin increases within the course of HFrEF possibly corresponding to disease progression. Contrarily to our assumption, renin trajectories were not associated with survival. Similarly, no differences could be identified in clinical characteristics between the groups besides the known physiologic triggers of renin.

In summary, it seems that renin, although it might bear some prognostic information for mortality or unplanned HF hospitalizations, is generally not a good predictor of HF risk. The single-point baseline measurement in our study was not convincingly associated with HF outcomes. Individual renin trajectories at 1 year, which was investigated by this study for the first time, did not provide any significant additional value. This is intriguing, given the significance of the RAS in HF pathophysiology and treatment.

The reasons for this can only be speculated. RAS is responsible for blood pressure and fluid homeostasis. Changes in these systems must be counteracted quickly. In contrast to these fast-action alterations of RAS and renin levels, detrimental effects on HF are believed to arise from a continuous overactivation. It might be that these changes obscure the comparably low variations in renin due to HF disease severity and thereby limit the value of plasma renin measurements. Also, this study might have been underpowered to detect significance. Another possibility, which is consistent with the fact that RAS blockade is an effective treatment while renin levels do not mirror disease progression, is that the full effect of RAS blockade is not explained by inhibiting unfavorable RAS actions of AngII. ACEi, for example, blocks the formation of AngII, thereby leading to high plasma AngI concentrations, which could be substrate for alternative RAS products as Ang1-7 which has counterregulatory cardioprotective effects [27]. ARBs block AT1R leaving AngII concentrations uninfluenced, which could bind to AngII type 2 receptor (AT2R) which similarly has opposite effects to AT1R [28]. Assuming that AngII actions are blocked sufficiently and that these alternate RAS mechanisms have a significant impact would mean that high renin would even be beneficial boosting the alternate pathways. It should not be forgotten that blocking the complete RAS by renin inhibitors as aliskiren has not been proven beneficial either, suggesting that some residual RAS activity is not detrimental but probably necessary [7]. New treatments should consider the RAS in its complexity. This might also explain the success of the multipeptidase inhibitor strategy where besides AngII inhibition, other vasoactive peptide systems related to RAS such as neprilysin inhibition by ARNI are used.

## 5. Conclusions

In contrast to the neurohumoral concept, almost one-third of patients with HFrEF under current GDMT show normal renin values. Renin levels, assessed as a single measurement, are not associated with increased mortality in this study. Assessing renin trajectories over one year adds no significant value. Nevertheless, renin may slowly rise during HF disease course reaching markedly elevated values at very severe disease where it might indicate terminal stages of HF. Novel treatments targeting the neurohumoral axis should consider the complexity of RAS and other vasoactive peptide systems.

## 6. Limitations

There are some limitations to this study. The single-center nature of this study at a tertiary care hospital may lead to a selection bias and limit generalization of the results. Although the study population was relatively large, more patients and longer FUP times would be desirable to more accurately estimate the real effect of renin on outcome.

## Figures and Tables

**Table 1:** Baseline characteristics of the patient cohort.

	Total (*N* = 505)	Renin tertile 1, range: 0.8–42.9 *μ*IU/ml (*N* = 169)	Renin tertile 2, range: 43–339 *μ*IU/ml (*N* = 168)	Renin tertile 3, range: 340–9564 *μ*IU/ml (*N* = 168)	*p* value
Active renin concentration (*μ*IU/ml), median [Q1; Q3]	123 [27.1; 621.0]	14.0 [6.2; 27.1]	124 [82.0; 214.0]	931 [626.0; 1840.0]	—
*Basic demographics*					
Age (years), median [Q1; Q3]	62 [52; 72]	64 [53; 72]	62 [52; 71]	61 [52; 72]	0.508
Gender, male, *n* (%)	390 (77.2%)	114 (67.5%)	136 (81.0%)	140 (83.3%)	<0.001
BMI (kg/m^2^), median [Q1; Q3]	27.3 [23.9; 31.4]	27.7 [23.6; 32.0]	26.4 [23.9; 30.1]	27.9 [24.8; 31.1]	0.416
Systolic blood pressure (mmHg), median [Q1; Q3]	125 [112; 140]	135 [126; 157]	125 [113; 140]	115 [105; 126]	<0.001
Heart rate (bpm), median [Q1; Q3]	71.0 [61.0; 81.0]	70.0 [60.0; 80.0]	72.0 [63.5; 82.0]	70.0 [61.5; 81.0]	0.602
NYHA class					0.872
NYHA I, *n* (%)	64 (12.9%)	22 (13.4%)	22 (13.3%)	20 (12.0%)	
NYHA II, *n* (%)	246 (49.7%)	85 (51.8%)	77 (46.7%)	84 (50.6%)	
NYHA III/IV, *n* (%)	185 (37.4%)	57 (34.8%)	66 (40.0%)	62 (37.3%)	

*Comorbidities*					
Ischemic HF, *n* (%)	277 (54.9%)	81 (47.9%)	95 (56.5%)	101 (60.1%)	0.069
Diabetes mellitus, *n* (%)	185 (36.6%)	57 (33.7%)	66 (39.3%)	62 (36.9%)	0.569
Hypertension, *n* (%)	275 (54.5%)	92 (54.4%)	94 (56.0%)	89 (53.0%)	0.861
Tumor, *n* (%)	67 (13.3%)	26 (15.4%)	17 (10.1%)	24 (14.3%)	0.324

*Medication and device therapy*					
Beta blocker, *n* (%)	467 (94.2%)	154 (94.5%)	158 (94.6%)	155 (93.4%)	0.870
Minimum 50% of TD BB, *n* (%)	364 (80.0%)	122 (78.7%)	126 (80.8%)	116 (80.6%)	0.884
RASi, *n* (%)					0.267
ACEi	238 (47.1%)	74 (43.8%)	87 (51.8%)	77 (45.8%)	
ARB	97 (19.2%)	28 (16.6%)	28 (16.7%)	41 (24.4%)	
ARNI	110 (21.8%)	40 (23.7%)	35 (20.8%)	35 (20.8%)	
Combination ACEi + ARB (based on MRA intolerance)	12 (2.4%)	5 (3.0%)	5 (3.0%)	2 (1.2%)	
No RASi	48 (9.5%)	22 (13.1%)	13 (7.7%)	13 (7.7%)	
Minimum 50% of TD RASi, *n* (%)	282 (72.5%)	97 (76.4%)	89 (66.9%)	96 (74.4%)	0.194
MRA, *n* (%)	375 (75.6%)	113 (68.9%)	121 (72.9%)	141 (84.9%)	0.002
Minimum 50% of TD MRA, *n* (%)	348 (97.2%)	106 (95.5%)	114 (97.4%)	128 (98.5%)	0.373
SGLT2i, *n* (%)	31 (19.5%)	5 (9.4%)	13 (25.0%)	13 (24.1%)	0.076
Minimum 50% of TD SGLT2i, *n* (%)	17 (65.4%)	3 (60.0%)	7 (70.0%)	7 (63.6%)	0.917
Ivabradin, *n* (%)	37 (10.1%)	6 (4.8%)	16 (13.2%)	15 (12.3%)	0.057
Loop diuretics, *n* (%)	239 (49.4%)	68 (42.0%)	78 (47.6%)	93 (58.9%)	0.009
ICD, *n* (%)	211 (43.1%)	56 (34.4%)	66 (41.3%)	89 (53.6%)	0.002
CRT, *n* (%)	140 (29.3%)	40 (24.7%)	44 (27.7%)	56 (35.7%)	0.085

*Laboratory parameters*					
Plasma aldosterone (pg/ml), median [Q1; Q3]	120 [72; 208]	99.0 [57.0; 158]	115 [72; 176]	160 [90; 288]	<0.001
NT-proBNP (pg/ml), median [Q1; Q3]	1780 [803; 3630]	1890 [878; 3720]	1930 [750; 3880]	1540 [764; 3310]	0.670
Creatinine (mg/dl), median [Q1; Q3]	1.2 [1.0; 1.6]	1.1 [0.9; 1.5]	1.1 [0.9; 1.6]	1.2 [1.0; 1.6]	0.530
BUN (mg/dl), median [Q1; Q3]	22.8 [16.9; 31.8]	20.2 [15.9; 29.2]	21.4 [15.7; 31.1]	25.4 [19.5; 36.4]	<0.001
Sodium (mmol/l), median [Q1; Q3]	140 [138; 142]	141 [139; 142]	140 [138; 142]	138 [136; 140]	<0.001
Potassium (mmol/l), median [Q1; Q3]	4.8 [4.5; 5.1]	4.7 [4.5; 5.0]	4.7 [4.4; 5.0]	4.9 [4.5; 5.3]	<0.001
Magnesium (mmol/l), median [Q1; Q3]	0.81 [0.75; 0.87]	0.81 [0.75; 0.86]	0.81 [0.75; 0.86]	0.83 [0.75; 0.90]	0.217
Albumin (g/l), median [Q1; Q3]	43.9 [41.3; 46.1]	43.4 [41.5; 45.7]	43.7 [41.3; 46.2]	44.4 [41.1; 46.5]	0.302
BChE (kU/l), median [Q1; Q3]	7.1 [5.8; 8.3]	7.0 [5.8; 8.1]	7.1 [6.0; 8.3]	7.2 [5.7; 8.4]	0.550
AST (GOT) (U/l), median [Q1; Q3]	24.0 [19.0; 30.0]	22.0 [19.0; 29.0]	24.0 [19.0; 30.0]	25.0 [20.0; 31.0]	0.767
ALT (GPT) (U/l), median [Q1; Q3]	23.0 [17.0; 32.8]	23.0 [16.0; 33.0]	21.0 [16.0; 31.0]	24.5 [18.0; 34.8]	0.369
GGT (U/l), median [Q1; Q3]	42.0 [24.0; 86.5]	38.0 [22.0; 73.0]	42.0 [24.0; 76.3]	48.0 [27.0; 114]	0.091
Bilirubin (mg/dl), median [Q1; Q3]	0.6 [0.4; 0.8]	0.5 [0.4; 0.7]	0.6 [0.4; 0.8]	0.6 [0.5; 0.9]	<0.001
Total cholesterol (mg/dl), median [Q1; Q3]	159 [126; 191]	173 [136; 196]	154 [121; 189]	154 [123; 182]	0.002
Hemoglobin (g/dl), median [Q1; Q3]	13.6 [12.3; 14.8]	13.6 [12.3; 14.6]	13.5 [12.2; 14.7]	13.8 [12.6; 14.9]	0.139
Thrombocytes (G/l), median [Q1; Q3]	220 [177; 263]	227 [188; 267]	209 [168; 257]	220 [178; 264]	0.345
Leucocyte count (G/l), median [Q1; Q3]	7.6 [6.4; 9.1]	7.4 [6.4; 8.8]	7.5 [6.2; 8.9]	7.9 [6.7; 9.4]	0.345
C-reactive protein (mg/dl), median [Q1; Q3]	0.3 [0.1; 0.7]	0.3 [0.2; 0.7]	0.3 [0.1; 0.7]	0.3 [0.1; 0.7]	0.970

ACEi: angiotensin-converting enzyme inhibitor; ARB: angiotensin receptor blocker; ARNI: angiotensin receptor-neprilysin inhibitor; BMI: body mass index; BChE: butyrylcholinesterase; CRT: cardiac resynchronization device; ICD: intracardiac defibrillator; MRA: mineralocorticoid receptor antagonist; SGLT2: sodium-glucose cotransporter-2. Continuous variables are given as median and 25^th^ and 75^th^ percentiles, and counts are given as numbers and percentages. For comparisons between groups, the Kruskal-Wallis rank sum test or Pearson's chi-squared test was used.

**Table 2:** Crude and multivariable Cox regression analysis assessing the impact of baseline and 1-year delta renin levels on all-cause mortality and unplanned HF hospitalization.

		Crude HR (95% CI)	*p* value	Model I HR (95% CI)	*p* value	Model II HR (95% CI)	*p* value	Model III HR (95% CI)	*p* value
All-cause mortality	Baseline renin (per 100 *μ*IU/ml increase)	1.01 (0.99-1.02)	0.414	1.01 (1.00-1.03)	0.062	1.02 (1.00-1.03)	0.037	1.01 (0.99-1.02)	0.313
	*Δ*-Renin % at 1 year (per 10% increase)	1.00 (1.00-1.00)	0.241	1.00 (1.00-1.00)	0.341	1.00 (1.00-1.00)	0.350	1.00 (1.00-1.00)	0.219

Unplanned HF hospitalization	Baseline renin (per 100 *μ*IU/ml increase)	1.01 (1.00-1.02)	0.015	1.01 (1.00-1.02)	0.019	1.01 (1.00-1.02)	0.014	1.01 (1.00-1.02)	0.066
	*Δ*-Renin % at 1 year (per 10% increase)	1.00 (1.00-1.00)	0.987	1.00 (1.00-1.00)	0.999	1.00 (1.00-1.00)	0.861	1.00 (1.00-1.00)	0.703

Model I: adjusted for age, sex, and body mass index. Model II: model I + additional adjustment for comorbidity type 2 diabetes, coronary artery disease, and hypertension. Model III: adjusted for NT-proBNP, NYHA class, and aldosterone.

**Table 3:** HF-related baseline characteristics for the renin evolution groups.

Characteristic	Normal-normal (*N* = 52)	Elevated-normal (*N* = 29)	Normal-elevated (*N* = 40)	Elevated-elevated (*N* = 164)	*p* value
Baseline renin (*μ*IU/ml), median [Q1; Q3]	10 [5; 19]	176 [84; 288]	27 [18; 35]	411 [156; 1058]	<0.001
*Basic demographics*					
Age (years), median [Q1; Q3]	65 [55; 74]	62 [57; 73]	56 [44; 70]	61 [51; 70]	0.109
Gender, male, *n* (%)	38 (73%)	23 (79%)	28 (70%)	135 (82%)	0.257
BMI (kg/m^2^), median [Q1; Q3]	27.3 [24.1; 32.2]	27.8 [24.1; 30.1]	25.9 [23.3; 31.8]	28.1 [24.4; 31.7]	0.818
Systolic blood pressure (mmHg), median [Q1; Q3]	148 [135; 170]	124 [115; 136]	130 [120; 145]	120 [110; 131]	<0.001
Heart rate (bpm), median [Q1; Q3]	69 [60; 82]	79 [66; 86]	74 [59; 79]	71 [62; 82]	0.351
NYHA class					0.796
NYHA I, *n* (%)	5 (9.8%)	4 (14%)	5 (12%)	23 (14%)	
NYHA II, *n* (%)	25 (49%)	13 (46%)	22 (55%)	83 (51%)	
NYHA III/IV, *n* (%)	21 (41%)	11 (39%)	13 (32%)	58 (35%)	

*Comorbidities*					
Ischemic HF, *n* (%)	26 (50%)	13 (45%)	19 (48%)	91 (55%)	0.617
Diabetes mellitus, *n* (%)	21 (40%)	15 (52%)	13 (32%)	65 (40%)	0.457
Hypertension, *n* (%)	30 (58%)	15 (52%)	18 (45%)	92 (56%)	0.589
Tumor, *n* (%)	8 (15%)	2 (6.9%)	3 (7.5%)	21 (13%)	0.542

*Medical and device therapy*					
Beta blocker, *n* (%)	51 (98%)	26 (90%)	35 (95%)	155 (96%)	0.319
ACEi, *n* (%)	23 (44%)	17 (59%)	20 (50%)	86 (52%)	0.622
ARB, *n* (%)	13 (25%)	8 (28%)	3 (7.5%)	43 (26%)	0.082
ARNI, *n* (%)	15 (29%)	5 (17%)	11 (28%)	30 (18%)	0.281
MRA, *n* (%)	36 (69%)	24 (83%)	30 (79%)	134 (83%)	0.204
SGLT2i, *n* (%)	0 (0%)	1 (14%)	1 (9.1%)	5 (16%)	0.444
Ivabradin, *n* (%)	4 (11%)	4 (19%)	2 (6.2%)	16 (14%)	0.519
Loop diuretics, *n* (%)	25 (50%)	13 (46%)	15 (39%)	84 (54%)	0.412
ICD, *n* (%)	18 (36%)	11 (38%)	17 (42%)	92 (59%)	0.011
CRT, *n* (%)	14 (28%)	7 (26%)	10 (26%)	54 (36%)	0.450

*Laboratory parameters*					
Plasma aldosterone (pg/ml), median [Q1; Q3]	119 [79; 158]	129 [104; 209]	87 [64; 143]	126 [75; 233]	0.022
NT-proBNP (pg/ml), median [Q1; Q3]	2760 [871; 5365]	1492 [469; 4022]	1841 [1010; 3472]	1764 [834; 3475]	0.289
Creatinine (mg/dl), median [Q1; Q3]	1.3 [1.0; 1.7]	1.3 [1.0; 1.8]	1.1 [0.9; 1.5]	1.1 [1.0; 1.5]	0.131
BUN (mg/dl), median [Q1; Q3]	27 [16; 34]	25 [19; 35]	18 [15; 27]	23 [18; 31]	0.059
Sodium (mmol/l), median [Q1; Q3]	141.0 [138.0; 142.0]	140.0 [137.0; 141.0]	141.0 [139.0; 142.2]	139.0 [137.0; 141.0]	<0.001
Potassium (mmol/l), median [Q1; Q3]	4.7 [4.6; 5.2]	5.2 [4.5; 5.4]	4.8 [4.4; 5.0]	4.8 [4.5; 5.1]	0.086
Magnesium (mmol/l), median [Q1; Q3]	0.81 [0.76; 0.88]	0.77 [0.71; 0.91]	0.80 [0.73; 0.84]	0.82 [0.75; 0.87]	0.472
Albumin (g/l), median [Q1; Q3]	43.8 [42.0; 45.7]	44.2 [39.8; 45.2]	43.4 [41.9; 45.4]	44.2 [42.4; 46.2]	0.672
BChE (kU/l), median [Q1; Q3]	7.3 [5.8; 8.1]	7.4 [6.0; 8.9]	6.7 [5.6; 8.4]	7.4 [5.8; 8.5]	0.768
Hemoglobin (g/dl), median [Q1; Q3]	13.5 [12.3; 15.0]	14.5 [12.9; 14.8]	13.6 [12.3; 14.5]	13.7 [12.6; 14.9]	0.334
C-reactive protein (mg/dl), median [Q1; Q3]	0.29 [0.16; 0.68]	0.28 [0.11; 0.59]	0.45 [0.16; 0.66]	0.27 [0.14; 0.64]	0.526

ACEi: angiotensin-converting enzyme inhibitor; ARB: angiotensin receptor blocker; ARNI: angiotensin receptor-neprilysin inhibitor; BMI: body mass index; BChE: butyrylcholinesterase; CRT: cardiac resynchronization device; ICD: intracardiac defibrillator; MRA: mineralocorticoid receptor antagonist; SGLT2: sodium-glucose cotransporter-2. Continuous variables are given as median and 25^th^ and 75^th^ percentiles, and counts are given as numbers and percentages. For comparisons between groups, the Kruskal-Wallis rank sum test or Pearson's Chi-squared test was used.

## Data Availability

Data used in this study are available from the corresponding author upon reasonable request.

## Abbreviations

ACEi: Angiotensin-converting enzyme inhibitors
Ang: Angiotensin
ARB: Angiotensin receptor blockers
ARC: Active plasma renin concentration
ARNI: Angiotensin receptor-neprilysin inhibitors
AT1R: AngII type 1 receptor
AT2R: AngII type 2 receptor
FUP: Follow-up
HFrEF: Heart failure with reduced ejection fraction
HR: Hazard ratio
GDMT: Guideline-directed medical therapy
LVEF: Left ventricular ejection fraction
MRA: Mineralocorticoid receptor antagonists
NT-proBNP: N-Terminal pro-B-type natriuretic peptide
NYHA: New York Heart Association
PRA: Plasma renin activity
RAS: Renin-angiotensin system
SGLT2i: Sodium-glucose cotransporter-2 inhibitors
TD: Target dose.

## Supplementary Materials

Supplementary Materials Supplementary Figure 1 shows crude hazard ratios (HR) with 95% confidence intervals (CI) per an increase of 100 *μ*IU/ml renin for all-cause mortality and unplanned HF hospitalizations stratified by the following subgroups: age, sex, eGFR, systolic blood pressure (SBP), and NYHA functional class. Supplementary Figure 2 shows the Kaplan-Meier plots of (i) survival and (ii) unplanned HF hospitalization free survival for patients grouped by <50%, >50%, and ±50% of change in renin concentration at 1 year compared to baseline and by renin elevation of more than 100 *μ*IU/ml.
