# Supplementary material for: Renin Trajectories and Outcome in Stable Heart Failure with Reduced Ejection Fraction (HFrEF) on Contemporary Therapy: A Monocentric Study from an Austrian Tertiary Hospital Outpatient Clinic
Source: J Renin Angiotensin Aldosterone Syst. 2023 Oct 31;2023:8883145. doi: 10.1155/2023/8883145 (PMC10630017; doi:10.1155/2023/8883145)

Supplemental Files:

Supplementary Figure 1. Subgroup analysis of baseline renin with all-cause mortality and unplanned HF hospitalizations. Crude HR with 95% CI are presented for subgroups of age, sex, eGFR, systolic blood pressure (SBP) and NYHA functional class.

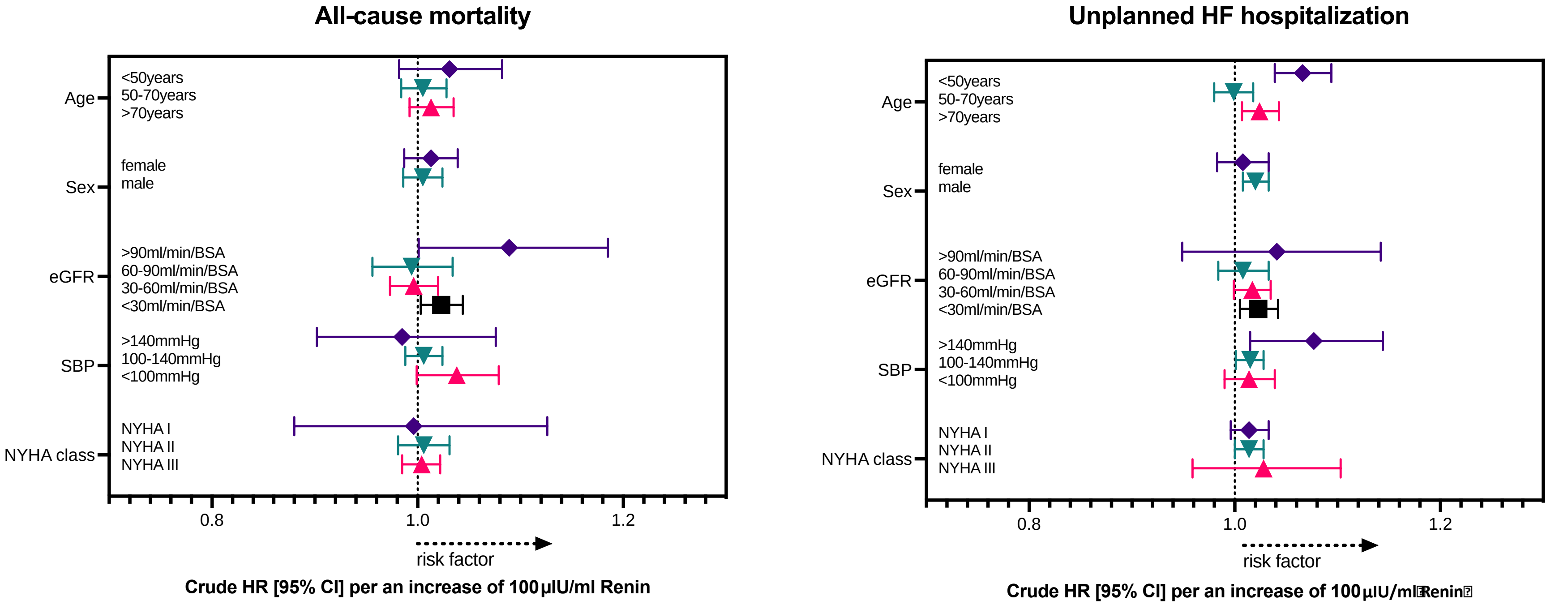

Supplementary Figure 2. Comparison of survival and unplanned HF hospitalization free survival of patients grouped by <50%, >50% and +-50% of change in renin concentration at 1-year compared to baseline and grouped by renin elevation of more than 100µIU/ml. The difference between groups was assessed by the log-rank test.

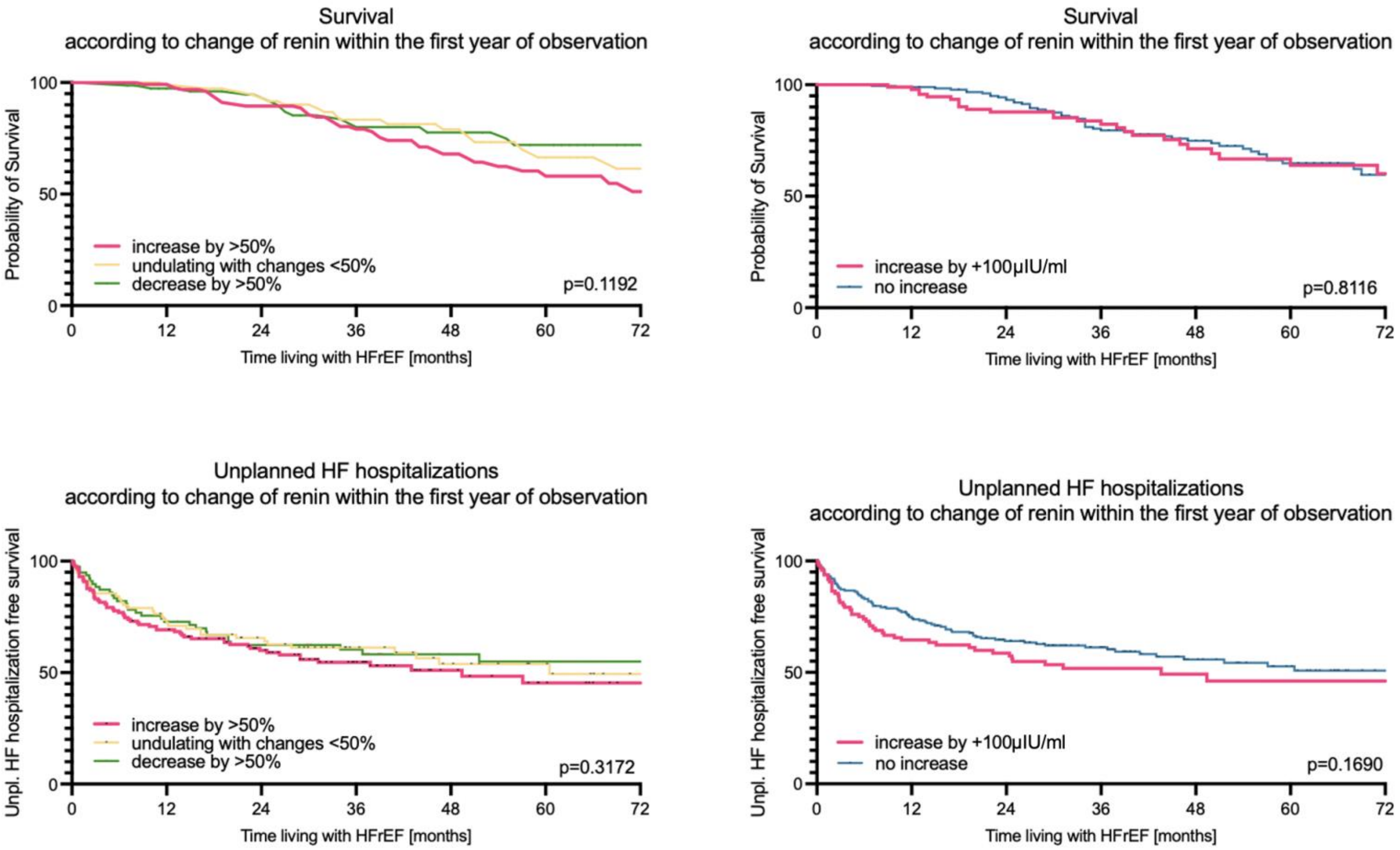

Supplement: Supplementary Materials — Supplementary Figure 1 shows crude hazard ratios (HR) with 95% confidence intervals (CI) per an increase of 100 μIU/ml renin for all-cause mortality and unplanned HF hospitalizations stratified by the following subgroups: age, sex, eGFR, systolic blood pressure (SBP), and NYHA functional class. Supplementary Figure 2 shows the Kaplan-Meier plots of (i) survival and (ii) unplanned HF hospitalization free survival for patients grouped by <50%, >50%, and ±50% of change in renin concentration at 1 year compared to baseline and by renin elevation of more than 100 μIU/ml. [file 8883145.f1.pdf]
